# *Shigella sonnei *biotype g carrying class 2 integrons in southern Italy: a retrospective typing study by pulsed field gel electrophoresis

**DOI:** 10.1186/1471-2334-6-117

**Published:** 2006-07-17

**Authors:** Caterina Mammina, Aurora Aleo, Cristina Romani, Antonino Nastasi

**Affiliations:** 1 Centre for Enteric Pathogens of southern Italy (CEPIM), Department of Hygiene and Microbiology "G. D'Alessandro", University, Via del Vespro 133, 90127 Palermo, Italy; 2 Department of Public Health, University, Via G.B. Morgagni 54, Florence, Italy

## Abstract

**Background:**

Emergence and global dissemination of multiresistant strains of enteric pathogens is a very concerning problem from both epidemiological and Public Health points of view. *Shigella sonnei *is the serogroup of *Shigella *most frequently responsible for sporadic and epidemic enteritis in developed countries. The dissemination is associated most often to human to human transmission, but foodborne episodes have also been described. In recent years the circulation of multiresistant strains of *S. sonnei *biotype g carrying a class 2 integron has been reported in many countries worldwide. In southern Italy a strain with similar properties has been responsible for a large community outbreak occurred in 2003 in Palermo, Sicily.

The objective of this study was to date the emergence of the biotype g strain carrying the class 2 integron in southern Italy and to evaluate the genetic heterogeneity of biotype g *S. sonnei *isolated throughout an extended interval of time.

**Methods:**

A total of 31 clinical isolates of *S. sonnei *biotype g identified in southern Italy during the years 1971–2000 were studied. The strains were identified at the serogroup level, characterized by biochemical tests and submitted to antimicrobial susceptibility testing. Molecular typing was performed by pulsed field gel electrophoresis (PFGE) after digestion of DNA by *Xba*I. Carriage of class 2 integrons was investigated by polymerase chain reaction (PCR) with specific primers and confirmed by restriction endonuclease analysis of amplicons.

**Results:**

The 15 isolates of *S. sonnei *biotype g identified in the decade 1971–1980 showed highly heterogeneous drug resistance profiles and pulsotypes. None of the isolates was simultaneous resistant to streptomycin and trimethoprim and none was class 2 integron positive. On the contrary, this resistance phenotype and class 2 integron carriage were very common among the 16 strains of biotype g identified in the following two decades. Moreover, all the more recent isolates, but one, showed closely related pulsotypes.

**Conclusion:**

Although our findings refer to a limited geographic area, they provide a snapshot of integron acquisition by an enteric pathogen responsible for several outbreaks in the years 2001–2003 in Italy. Molecular typing, indeed, suggests that the emergence of biotype g class 2 integron carrying *S. sonnei *in southern Italy should be backdated to at least the late 1980s. In the following decades, the circulation of biotype g appears to be sustained by multiresistant highly related strains. Similar trend are described in several countries, but the questions about mechanism of emergence and worldwide spread of this pathogen remain open.

## Background

The issue of emergence and global dissemination of multiresistant strains of enteric pathogens is of greatest interest from both epidemiological and prevention/control points of view. Whereas emergence is unavoidable, the successful and persistent dissemination through widest geographic areas of a resistant strain needs to be supported by the selective pressure due to the large use of antibiotics [1].

*Shigella sonnei *is the major agent of shigellosis in developed countries, where sanitation and environmental hygiene have largely interrupted the preferential transmission routes of other serogroups of *Shigella*, like *dysenteriae*, *boydii *and *flexneri *[2]. In recent years, several reports on *S. sonnei *epidemiology from different countries have associated the epidemic circulation of this organism to a well defined strain, characterized by some properties: lack of rhamnose fermentation (biotype g), a resistant phenotype streptomycin-sulfonamide-trimethoprim and, less consistently, tetracycline, a distinct *Xba*I pulsotype and the presence of a 2.2 Kbp class 2 integron [3-9]. Although a direct comparison between strains from different countries has not been reported until now, the findings by various authors seem to be suggestive of a worldwide occurrence of cases due to a pandemic strain of *S. sonnei *[7].

In southern Italy, during the period May-December 2003 a large community outbreak of shigellosis involved children and young adults in Palermo, Sicily. Phenotypic and genetic characterization of the isolates identified at the Centre for Enteric Pathogens of southern Italy (CEPIM) showed that *S. sonnei *biotype g carrying class 2 integrons and showing a peculiar pulsotype was responsible for this event [7].

During the previous decades, isolation of *S. sonnei *biotype g in southern Italy had a very irregular trend: in the decade 1971–'80 it accounted for 17.5% (72 of 412) of the isolates of *S. sonnei*, whereas in the next decade 1981–'90 for 3.5% (26 of 779) and in the years 1991–2000 for 71.4% (40 of 56) [10]. This last period was characterized by a striking decline in cases of *S. sonnei *infection, most frequently occurring within patent epidemic events: indeed, 28 of the 40 biotype g isolates were from a community outbreak occurring in 1991 in Palermo, Sicily.

The objective of this study was to date the emergence of the biotype g strain carrying the class 2 integron in southern Italy and to evaluate by pulsed field gel electrophoresis (PFGE) the genetic heterogeneity of biotype g *S. sonnei *isolated throughout an extended interval of time.

## Methods

### Bacterial strains

A total of 31 clinical isolates of *S. sonnei *biotype g identified in southern Italy during the years 1971–2000 were studied. The sample was chosen on the basis of available epidemiological information, that suggested that a high proportion of the isolates in the period 1981–2000 clustered in three groups: indeed, 12 isolates were identified in Brindisi, Apulia, in October 1989, 12 in Brindisi also, but in January 1990, and 28 in Palermo, Sicily, in October-November 1991. However, except for the isolates from Palermo, the temporal clusters had not been formally recognized as epidemic events by the Public Health services. The 31 strains, then, included: 15 randomly chosen isolates out of 72 identified in the years 1971–1980; eight of 26 isolates identified in the years 1981–1990, all belonging to the two clusters of cases of Brindisi in 1989 and 1990; eight of 40, seven of which apparently sporadic, identified in the years 1991–2000.

The strains had been identified at the serogroup level. and characterized by biochemical tests at the CEPIM by previously described methods [10]. Antimicrobial susceptibility testing was performed by the disk diffusion method as recommended by the National Committee of Clinical Laboratory Standards [11]. The following antimicrobial agents were tested (the disk content is indicated in parentheses): ampicillin, Ap (10 μg), chloramphenicol, Cm (30 μg), streptomycin, Sm (10 μg), sulfonamides, Su (300 μg), tetracycline, Tc (30 μg) trimethoprim, Tp (5 μg), ciprofloxacin, Cp (5 μg), kanamycin, Km (30 μg) and nalidixic acid, Na (30 μg). *Escherichia coli *ATCC 25922 was used as a control. The disks were obtained from Oxoid (Basingstoke, United Kingdom).

### Pulsed Field Gel Electrophoresis (PFGE)

*S. sonnei *isolates were grown in 3 ml Brain Hearth Infusion (BHI) broth at 37°C overnight. The cells were pelleted, washed and resuspended in 1 ml of 75 mM NaCl, 25 mM EDTA. Then they were mixed with the same volume of 1.2% agarose gel (pulsed-field grade; Bio-Rad, Hercules, CA, USA) and poured into the slots of a plastic mould (CHEF DRII; Bio-Rad). The agarose plugs obtained were transferred into the lysis buffer (50 mM Tris-HCl, pH 7.2, 50 mM EDTA, 1.0% N-laurylsarcosine and 0.1 mg/ml proteinase K) and cells were lysed for 16–20 h at 55°C. Then, the agarose plugs were washed five times for 30 min with washing buffer (10 mM Tris-HCl, pH 8.0, 1 mM EDTA) with gently shaking and stored in the same buffer at 4°C.

A 1-mm slice of each agarose plug was equilibrated in 100 μl restriction buffer for 30 min and digested at 37°C for 18 h with 50 units XbaI (Promega, Madison, WI, USA). After digestion, genomic DNA was separated in 1% agarose gel with a contour-clamped homogeneous electric field apparatus (CHEF-DRIII, Bio-Rad Laboratories). The conditions for electrophoresis were 6 V/cm for 21 h with the pulse time increasing from 5 to 40 s. The strain of *Salmonella *serotype Braenderup H9812 restricted with *Xba*I was used as molecular weight standard. Strain H9812 was kindly provided by the National Reference Centre for Enteric Pathogens at Istituto Superiore di Sanità, Rome, Italy. PFGE patterns were initially visually assessed and interpreted by using the criteria established by Tenover *et al*. [12]. Computer-assisted analysis of the PFGE banding patterns was performed with the Diversity Database software (Bio-Rad Laboratories).

### Integron analysis

To detect the class 2 integrons polymerase chain reaction (PCR) was performed with the specific primer pair hep74 (5'-CGGGATCCCGG AGGCATGCACGATTTGTA-3') and hep51 (5'-ATGCCATCGCAAGTACGAG-3'). Primer hep74 binds to *attI*2, and hep51 binds to *orfX*, situated at the right end of the cassette region within transposon Tn7 [13]. Tn7-containing strain *Escherichia coli *K-12 J62 (ColE1::Tn7) was used as positive control strain [6,7]. To each reaction mixture was added 100 ng of template DNA, 1.5 mM MgCl2, each deoxynucleoside triphosphate at a concentration of 200 μM, 5 μl of 10× PCR buffer (50 mM Tris-HCl [pH 8.0], 100 mM NaCl, 0.1 mM EDTA, 1 mM dithiothreitol, 50% glycerol, 1% Triton X-100), 25 pmol of each primer, and 2.5 U of *Taq *DNA polymerase (Promega) in a final reaction volume of 50 μl. Amplification was performed by using the following temperature profile: pre-denaturation at 94°C for 10 min, followed by 35 cycles of 94°C for 1 min, 55°C for 1 min, and 72°C for 5 min, with a final extension step at 72°C for 5 min. The amplified DNA products were analyzed by conventional 1% agarose gel electrophoresis in 1× TBE buffer and stained with ethidium bromide. The similarity among the 2.2-kb amplicons of the *E. coli *reference strain and *S. sonnei *isolates was investigated by restriction analysis with the endonucleases *Ava*I, *Hin*cII and *Hin*fI [7].

## Results

### *S. sonnei *isolates identified in the period 1971–1980

**Figure 1:**
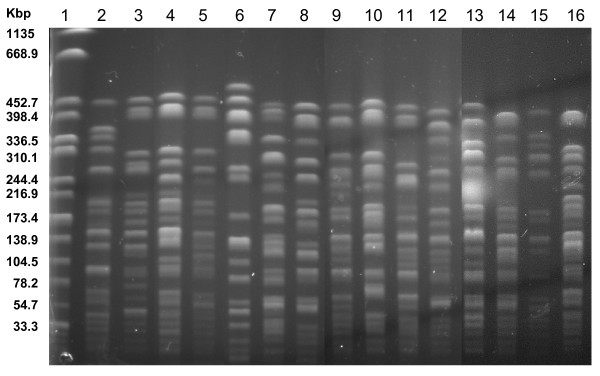
**PFGE profiles of the *Shigella sonnei *biotype g isolates, 1971–1980**. Lanes: 1, *Salmonella *Braenderup H9812 (molecular weight standard); 2–16, *S. sonnei *biotype g isolates: 2, Catanzaro, Calabria, 1979; 3, Palermo, Sicily, 1972; 4, Palermo 1975; 5, Palermo 1971; 6, Catanzaro 1979; 7, Palermo 1972; 8, Palermo 1973; 9, Catanzaro 1979; 10, Palermo 1972; 11, Cosenza, Calabria, 1979; 12, Palermo 1971; 13, Palermo 1973; 14, Catanzaro 1979; 15, Palermo 1971; 16, Palermo 1975.

Six different susceptibility patterns were identified, with five isolates being susceptible to the tested panel of antibacterial drugs. None of the isolates was simultaneous resistant to streptomycin and trimethoprim. PCR for class 2 integrons tested negative for all isolates (Table 1). Fifteen different unrelated or poorly related pulsotypes were identified: except for two virtually identical profiles (Fig. 1, lanes 2 and 15), only one pair of isolates, indeed, showed less than seven different bands (Fig. 1, lanes 3 and 5), whereas all the remaining were heterogeneous (Fig. 1).

**Table 1:** Characteristics of the isolates included in the study

Years	Number of isolates	Susceptibility pattern	Class 2 integron
1971–1980	5	Susceptible	Negative
	4	SmSuTc	"
	3	SmSu	"
	1	SmSuCmTc	"
	1	SmSuCm	"
	1	Su	"
1981–1990	7	SmSuTcTp	Positive
	1	ApSmSuTcTp	Positive
1991–2000	5	SmSuTcTp	Positive
	1	SmSuTcTp	Negative
	2	SmSuTp	Positive

Ap = ampicillin; Cm = cloramphenicol; Sm = streptomycin; Su = sulfonamides; Tc = tetracycline; Tp = trimethoprim

### *S. sonnei *isolates identified in the period 1981–1990

**Figure 2:**
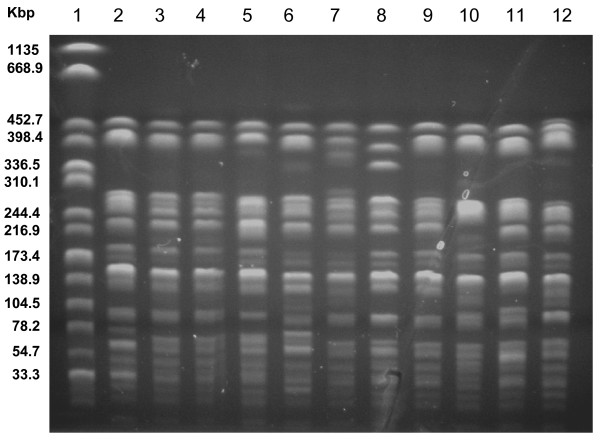
**PFGE profiles of the *Shigella sonnei *biotype g isolates, 1981–2000**. Lanes: 1, *Salmonella *Braenderup H9812 (molecular weight standard); 2–12, *S. sonnei *biotype g isolates: 2, epidemic strain, Palermo, Italy, 2003; 3, Brindisi, Apulia, 1989; 4, Brindisi 1990; 5, Palermo 1991; 6, Palermo 1994; 7, Palermo 1995; 8, Reggio Calabria, Calabria, 1991; 9, Palermo 1994; 10; Palermo 1995; 11, Messina, Sicilia, 1993; Palermo 1996.

The eight strains exhibited two different resistance patterns, differing only by susceptibility to ampicillin. A SmSuTpTc pattern was identified in seven strains (Table 1). A class 2 integron was present in all isolates, that shared also the same pulsotype (Fig. 2). The sequence similarity of the 2.2-kb amplicons obtained from *S. sonnei *isolates and the Tn7 containing reference strain of E. coli was confirmed by identity of *Ava*I, *Hin*cII and *Hin*fI restriction patterns.

### *S. sonnei *isolates identified in the period 1991–2000

Two different susceptibility patterns, differing by susceptibility to tetracycline, were identified within these eight strains. All strains, but one identified in Palermo, Sicily, during 1995, were positive for class 2 integrons (Table 1). Restriction endonuclease analysis confirmed the similarity of the amplicons obtained from these further isolates and the reference strain. All strains, but one (Fig. 2, lane 8) identified from an apparently sporadic case occurred in 1991 in Reggio Calabria, Calabria, exhibited closely related pulsotypes (Fig. 2).

A phenotypic and genetic comparison between the isolates identified in the period 1981–2000 and the outbreak strain of Palermo, 2003, showed a close relationship: indeed, 11 of 16 strains had identical susceptibility patterns and a class 2 integron and showed highly related PFGE patterns (Fig. 2).

## Discussion

Shifting of serogroups of *Shigella *from *flexneri *to *sonnei *and the steady progression from high endemicity to low endemicity and, eventually, an epidemiological mix of sporadic cases and infrequent outbreaks parallel the evolution from developing to developed countries [2]. In our geographic area, this trend has been observed in the last decades in association with an additional epidemiological aspect: the endemic phase appeared to be associated with biotype a, whereas the more recent epidemic clusters to biotype g [7,10]. Other European countries have noticed a similar behaviour of *Shigella *and in many other regions worldwide biotype g or rhamnose negative strains could be included among the emergent organisms [4].

Our findings show that strains of *S. sonnei *biotype g circulating in southern Italy in the first decade under study were heterogeneous by PFGE and drug resistance pattern and class 2 integron free. Within isolates of the following decades, two features turned to be a consistent and distinctive genetic marker: carriage of integrons and a substantial homogeneity of PFGE profiles. A subsequent step may be recognized in the outbreak phase, when closely related, class 2 integron-carrying strains of biotype g have been identified as the etiologic agent of a communitywide epidemic event occurring in 2003 in Palermo, Sicily [7]. Lombardy also, a region of Northern Italy, experienced in 2001 three classic outbreaks in two daycare centres and a small community, that were attributed to a highly related strain of *S. sonnei *biotype g [7].

Simultaneous emergence in widespread different areas of infectious agents having humans as principal reservoir is more difficult to justify than that of zoonotic organisms, especially those preferentially transmitted through the food chain. However, several foodborne outbreaks caused by *S. sonnei *and prominently vehicled by fresh produce have been reported worldwide [14-16]. Moreover, *S. sonnei *has been proved to be able to survive in food also in apparently hostile ecological conditions [17]

The question whether spread in many countries of *S. sonnei *biotype g could be associated to a common food vehicle or the emergence from an endemic pool of bacterial strains and subsequent dissemination of more biologically fit organisms cannot be readily answered. A great contribute can be provided by collaborative molecular epidemiological studies adopting standardized methodologies and by more efficient international surveillance networks.

## Conclusion

Our findings prove that emergence of biotype g, class 2 integron carrying *S. sonnei *should be backdated to at least the late 1980s, when a circulation of closely related strains is confirmed also by the substantial homogeneity of PFGE profiles in comparison with the striking heterogeneity of the decade 1971–'80. The hypothesis is, of course, conservative because of the limits of our retrospective study due to the inherent poor sensitivity of passive surveillance systems and the restricted availability of viable strains for analysis.

In our geographic area, like in many countries, shifting to streptomycin or trimethoprim as the elective therapeutic option was largely adopted after the emergence in the 1950s of sulfonamide resistant strains of *Shigella *spp. Hence, in the course of the three decades under study, the presence of a class 2 integron might have positively driven the successful epidemiological evolution and spread of a streptomycin-trimethoprim resistant lineage.

## Competing interests

The author(s) declare that they have no competing interests.

## Authors' contributions

CM conceived the study, carried out the drug resistance and integron analysis and drafted the manuscript. AA and CR performed search for archival strains, biochemical typing and PFGE. AN participated in study design and coordination and helped to draft the manuscript. All authors read and approved the final manuscript.

## Pre-publication history

The pre-publication history for this paper can be accessed here:


